# Metformin-Inducible Small Heterodimer Partner Interacting Leucine Zipper Protein Ameliorates Intestinal Inflammation

**DOI:** 10.3389/fimmu.2021.652709

**Published:** 2021-06-15

**Authors:** SeungCheon Yang, Jin-Sil Park, Sun-Hee Hwang, Keun-Hyung Cho, Hyun Sik Na, JeongWon Choi, Jooyeon Jhun, Seung-Jun Kim, Bo-In Lee, Sung-Hwan Park, Mi-La Cho

**Affiliations:** ^1^ The Rheumatism Research Center, Catholic Research Institute of Medical Science, College of Medicine, The Catholic University of Korea, Seoul, South Korea; ^2^ Department of Biomedicine & Health Sciences, College of Medicine, The Catholic University of Korea, Seoul, South Korea; ^3^ Division of Gastroenterology, Department of Internal Medicine, College of Medicine, Seoul St. Mary’s Hospital, The Catholic University of Korea, Seoul, South Korea; ^4^ Division of Rheumatology, Department of Internal Medicine, Seoul St. Mary’s Hospital, College of Medicine, The Catholic University of Korea, Seoul, South Korea; ^5^ Department of Medical Lifescience, College of Medicine, The Catholic University of Korea, Seoul, South Korea

**Keywords:** SMILE, AMPK, Foxp3, IL-17, fibrosis

## Abstract

Small heterodimer partner interacting leucine zipper protein (SMILE) is an orphan nuclear receptor and a member of the bZIP family of proteins. We investigated the mechanism by which SMILE suppressed the development of inflammatory bowel disease (IBD) using a DSS-induced colitis mouse model and peripheral blood mononuclear cells (PBMCs) from patients with ulcerative colitis (UC). Metformin, an antidiabetic drug and an inducer of AMPK, upregulated the level of SMILE in human intestinal epithelial cells and the number of SMILE-expressing cells in colon tissues from DSS-induced colitis mice compared to control mice. Overexpression of SMILE using a DNA vector reduced the severity of DSS-induced colitis and colitis-associated intestinal fibrosis compared to mock vector. Furthermore, SMILE transgenic mice showed ameliorated DSS-induced colitis compared with wild-type mice. The mRNA levels of SMILE and Foxp3 were downregulated and SMILE expression was positively correlated with Foxp3 in PBMCs from patients with UC and an inflamed mucosa. Metformin increased the levels of SMILE, AMPK, and Foxp3 but decreased the number of interleukin (IL)-17–producing T cells among PBMCs from patients with UC. These data suggest that SMILE exerts a therapeutic effect on IBD by modulating IL-17 production.

## Introduction

Inflammatory bowel disease (IBD) is a chronic, progressive, and relapsing inflammatory disorder of the gastrointestinal tract related to multiple genetic and environmental factors (1). The two major clinical subtypes of IBD are ulcerative colitis (UC), which features mucosal inflammation of the large intestine only, and Crohn’s disease (CD), which involves transmural chronic inflammation of the entire gastrointestinal tract (2). Characterized by increased deposition of extracellular matrix factors, intestinal fibrosis is a frequent complication leading to dysfunctional wound healing and colonic wall thickening (3, 4).

The pathogenesis of IBD is unclear; however, intestinal mucosal dysfunction and uncontrolled immune responses mediated by intestinal epithelial cells and immune cells are important factors in its development (5–7). Interleukin (IL)-17-producing T helper (Th17) cells are main players in the development of IBD (8). Th17 cells infiltrate inflamed colonic lamina propria and disease progress is associated with an increased serum IL-17 level in colitis mice (9). IL-17 deficiency suppresses the development of inflammatory colitis induced by dextran sodium sulfate (DSS) (10). Regulatory T (Treg) cells secrete the anti-inflammatory cytokine IL-10 and suppress the functions of other immune cell types, particularly Th17 cells (11, 12). B cells also secrete IL-17 in response to pathogens (13) and IL-10-producing B (B10) cells modulate the immune response during inflammation (14).

Small heterodimer partner-interacting leucine zipper protein (SMILE) belongs to the cAMP response element-binding and activating transcription factor (CREB/ATF) family of basic region-leucine zipper (bZIP) transcription factors and binds to DNA as a homodimer. It has two alternative translation-derived isoforms, SMILE-L (CREBZF; long form of SMILE) and SMILE-S (Zhangfei; short form of SMILE) (15, 16). SMILE is a transcriptional coregulator of estrogen receptor (ER), ER-related receptor γ, constitutive androstane receptor, and glucocorticoid receptor (16–18). However, the role of SMILE in IBD has not yet been elucidated.

We investigated the effect of SMILE on DSS-induced colitis in mice. Overexpression of SMILE ameliorated the progression of DSS-induced colitis. SMILE modulated mTOR-STAT3 signaling and reduced the production of proinflammatory cytokines, such as IL-6, IL-1β, and IL-17, in the colon. Metformin increased the levels of SMILE, AMPK, and Foxp3 but decreased the number of IL-17-producing T cells in PBMCs from patients with UC and an inflamed mucosa.

## Materials and Methods

### Animals

C57BL/6 mice were purchased from Orient Bio Inc. (South Korea). The animals were maintained under specific pathogen-free conditions at the Institute of Medical Science of the Catholic University of Korea and fed standard mouse chow (Ralston Purina, USA) and housed five per cage under controlled temperature (21–22°C) and light (12/12 h light/dark cycle) conditions with access to gamma lay sterilized diet (Harlan Laboratories, USA) and autoclaved R/O water. Specific pathogen-free condition is monitored according to the category of International Council for Laboratory Animal Science (ICLAS) and Ministry of Food and Drug Safety of Korea, and checked Ectromelia virus(mouse pox), Lymphocytic choriomeningitis virus(LCMV), Mouse hepatitis virus(MHV), Sendai virus, Hantavirus, Minute virus of mice(MVM), Mouse adenovirus(MAV), Mouse cytomegalovirus(MCMV), Pneumonia virus of mice(PVM), Reo3, Rotavirus(Epizootic diarrhea of infant mice, EDIM), Theiler’s murine encephalomyelitis virus(TMEV/GDVII), Lactate dehydrogenase-elevating virus, K virus, Mouse norovirus(MNV), Mouse parvovirus(MPV), Mouse thymic virus(MTV), Polyoma virus, *Citrobacter rodentium, Clostridium piliforme(Tyzzer’s disease), Mycoplasma pulmonis, Pateurella pneumotropica, Salmonella spp, Pseudomonas aeruginosa, Helicobacter hepaticus, Staphylococcus aureus, Klebsiella oxytoca, Klebsiella pneumoniae, Corynebacterium bovis*, Pneumocystis murina, Pinworm, Intestinal protozoa, Ectoparasites. All experimental procedures were provided in accordance with the Laboratory Animals Welfare Act, the Guide for the Care and Use of Laboratory Animals and the Guidelines and Policies for Rodent experiment provided by the IACUC (Institutional Animal Care and Use Committee) in school of medicine, The Catholic University of Korea. To generate SMILE transgenic mice, a pcDNA3.0 vector was constructed containing a cytomegalovirus promoter. The SMILE fragment was synthesized by GenScript Corporation (USA), with codon optimization for expression in mammalian cells. The open reading frame originated in mice. Transgenic mice overexpressing SMILE were generated on a C57BL/6 background by transgene microinjection and maintained at Macrogen Inc. (South Korea). SMILE transgenic founder mice were mated to C57BL/6 mice and were crossed for 10 generation. The presence of the transgene in the founders was confirmed by polymerase chain reaction (PCR) using genomic DNA extracted from the tail.

### Patients

The subjects (18 patients) were diagnosed with UC. Of the 18 patients, 9 were severe and 9 were remission, of which 10 were male and 8 were female. The clinical, endoscopic, and histologic findings of the subjects were consistent with those of UC. Colonic mucosal tissue was obtained from the rectum during surveillance colonoscopy and peripheral blood was sampled. The patients provided informed written consent to participate in the study. This study was approved by the Institutional Review Board of Seoul St. Mary’s Hospital (XC18TEDI0027) and performed in accordance with the Helsinki II Declaration.

### Cell Culture

HT-29 human colon cells (ATCC, USA) were maintained in Roswell Park Memorial Institute (RPMI) 1640 medium (Gibco BRL, USA) containing 10% fetal bovine serum (FBS) (Gibco BRL, USA). Cells were stimulated with or without metformin (5 mM) for 48 h and the lysates were subjected to western blotting. PBMCs from patients with UC were isolated from buffy coats in heparinized blood samples by Ficoll-Hypaque (Amersham Biosciences, UK) density-gradient centrifugation. PBMCs were maintained in RPMI medium containing 10% FBS. PBMCs were stimulated with or without metformin (1 mM) for 72 h and mRNA levels and the number of Th17 cells were examined by real-time PCR and flow cytometry, respectively.

### DSS-Induced Colitis and Drug Administration

Colitis was induced in C57BL/6 male mice by oral administration of 3% DSS (MP Biomedicals, USA) *via* drinking water for 5 days. The mice were randomly separated into two groups (n = 5 per group). For metformin experiments, mice were orally administrated metformin (50 mg/kg) or saline (200 μL) as a control, daily after colitis induction. Mice weights were recorded daily. For SMILE overexpression experiments, SMILE cDNA fragment was cloned into the pcDNA3.0 vector between *EcoR*I and *Kpn*I. Escherichia coli containing the SMILE overexpression vector were incubated in Luria–Bertani highsalt broth (Duchefa Biochemie, Netherlands) for 16 h at 37 °C and 160 rpm. The cells were harvested by centrifugation, the SMILE overexpression vector purified using a NucleoBond Xtra Maxi EF Kit (Macherey-Nagel, Germany). Mice were intravenously injected with 100 μg of SMILE overexpression vector or control vector in 1 mL of saline, 1 day before colitis induction. Additional injections were given on the 4 and 9 days after colitis induction (approval number CUMC 2020-0139-01). SMILE TG mice received 3% DSS for 5 days. The body weight of the mice was measured daily (approval number: CUMC 2020-0207-01).

### Western Blotting

Cells were lysed in RIPA lysis and extraction buffer containing Halt protease inhibitor cocktail (Thermo Scientific, USA). Lysates were centrifuged at 14,000 rpm for 15 min at 4°C. Protein concentration was determined using a Pierce BCA Protein Assay Kit (Thermo Scientific, USA). Proteins were resolved by sodium dodecyl sulfate-polyacrylamide gel electrophoresis and transferred to membranes (GE Healthcare, USA). The membranes were incubated with antibodies against STAT3, phosphorylated STAT3 Y705 (Cell Signaling, USA), SMILE, GAPDH (Abcam), and β-actin (Santa Cruz Biotechnology, USA). Hybridized bands were detected by enhanced chemiluminescence (Thermo Scientific, USA) on X-ray film (AGFA, Belgium). Western blotting was performed using the SNAP i.d. Protein Detection System.

### Flow Cytometry

The mesenteric lymph nodes (MLNs) were removed from the mice and single cells were isolated and immunostained using fluorescently conjugated antibodies against CD4, IL-17, CD25, Foxp3, CD19, CD5, CD1d, and IL-10. Prior to intracellular staining, cells were stimulated for 4 h with phorbol 12-myristate 13-acetate (PMA) (25 ng/mL) and ionomycin (250 ng/mL) in the presence of GolgiStop™ (BD Biosciences, USA). Intracellular staining was performed using a BD Cytofix/Cytoperm Plus Fixation/Permeabilization Kit and BD Golgistop Kit (BD Biosciences, USA). The transcription factor Foxp3 was stained using a Foxp3/Transcription Factor Staining Kit (eBioscience, USA) according to the manufacturer’s instructions.

PBMCs from patients with UC were restimulated with PMA (25 ng/mL) and ionomycin (250 ng/mL) in the presence of GolgiStop™ for 4 h and immunostained for CD4, IL-17, CD25, and Foxp3 (eBiosciences, USA). Flow cytometry was performed using a cytoFLEX Flow Cytometer (Beckman Coulter, USA).

### Histopathological Analysis

Colon tissue was fixed in 10% (v/v) neutral-buffered formalin (Sigma-Aldrich, USA) and embedded in paraffin. Next, 5 μm sections were prepared and stained with hematoxylin and eosin (H&E). A histological scoring system was used to evaluate the severity of colitis. The sections were also assessed, using scales of 0–3, for loss of epithelium (0, no loss; 1, 0–5% loss; 2, 5–10% loss; and 3, > 10% loss of epithelium), crypt damage (0, no damage; 1, 0–10% loss of crypts; 2, 10–20% loss of crypts; and 3, > 20% loss of crypts), depletion of goblet cells (0, none; 1, mild; 2, moderate; and 3, severe), and infiltration of inflammatory cells (0, none; 1, mild; 2, moderate; and 3, severe). At least three sections from each colon were analyzed. Biopsy specimens of patients with UC were graded using the Mayo scoring system for Assessment of Ulcerative Colitis Activity (19–21). The Mayo score ranges from 0 to 12, with higher scores indicating more severe disease. We also assessed stool frequency, rectal bleeding, and partial Mayo score and performed a physician’s assessment.

### Immunohistochemistry

Formalin-fixed colon sections were paraffin-embedded and 5 μm sections were deparaffinized using xylene and dehydrated in an alcohol series. At least four sections from each colon were analyzed. The sections were treated with proteinase K in Tris-ethylenediaminetetraacetic acid buffer for antigen retrieval and washed in 1 × phosphate-buffered saline (PBS; pH 7.5). Endogenous peroxidase activity was quenched with methanol and 3% H_2_O_2_. Immunohistochemistry (IHC) was performed using the Envision Detection System (Dako, Denmark). The tissues were incubated with primary anti-SMILE, anti-AMPKα, anti-IL-1β, anti-IL-17, anti-STAT3, anti-IL-6, anti-α-SMA, anti-collagen-1 (Abcam, UK) and anti-mTOR (Cell Signaling, USA) antibodies overnight at 4°C followed by a horseradish peroxidase-conjugated secondary antibody for 30 min. The final color product was developed using 3,3-diaminobenzidine (Dako, Denmark). The sections were counterstained with Mayer’s hematoxylin and examined by photomicroscopy (Olympus, Japan).

### Real-Time Polymerase Chain Reaction

Total RNA was extracted using TRI Reagent (Molecular Research Center, USA) and cDNA was synthesized with the Dyne First-Strand cDNA Synthesis Kit (Dyne Bio, South Korea) according to the manufacturer’s protocol. mRNAs were quantified using the StepOnePlus™ Real-Time PCR system (Applied Biosystems, USA) with SensiFAST SYBR Hi-ROX (Bioline, USA). mRNA levels were normalized to that of βactin. The following primers were used: *β-actin*, 5'–GGACTTCGAGCAAGAGATGG-3' and 5'-TGTGTTGGGGTACAGGTCTTTG-3'; *SMILE*, 5'–GACCTGCTGCAAAGGCTGTTA-3' and 5'-CTGGTTGTTGTCGTTACCGCT-3'; *PRKAA*, 5'–ACACCCAACCTCAGACAAGG-3' and 5'-ATCACGTGACGGTGGTTACA-3'; and *FOXP3*, 5'–CACTGCCCCTAGTCATGGT-3' and 5'-GGAGGAGTGCCTGTAAGTGG-3’.

### Statistical Analysis

Statistical analysis was performed using Prism (GraphPad Software, USA). Normally distributed continuous data were analyzed by parametric Student’s *t*-test. Differences in means among groups were subjected to analysis of variance followed by Bonferroni *post hoc* test. Values are means ± standard error of the mean (SEM). A value of *p* < 0.05 was indicative of statistical significance.

## Results

### Metformin Increases the SMILE Level in Colon Tissue

**Figure 1:**
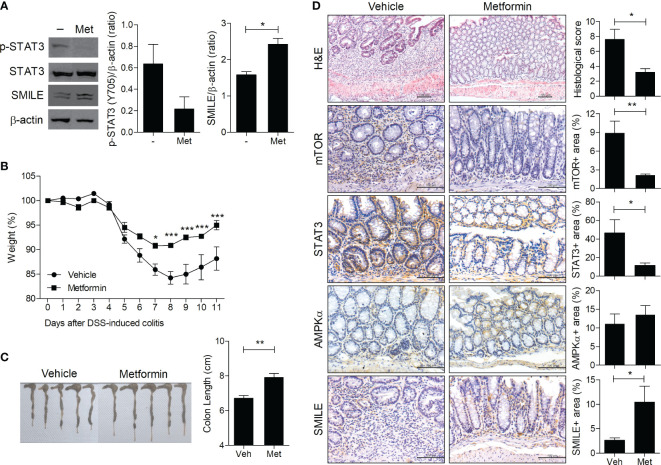
Metformin upregulates SMILE in DSS-induced colitis. **(A)** Western blots of STAT3, p-STAT3, and SMILE expression in metformin (5 mM)-treated and untreated HT-29 cells for 48 h, followed by densitometric analysis. **(B)** Metformin (50 mg/kg) was administered with DSS. Body weight is expressed as a percentage (n = 5). **(C)** Colon length of mice with DSS-induced colitis on day 11. Macroscopic images of the colon are shown, and the colon length was measured. **(D)** Hematoxylin and eosin (H&E) staining and IHC with anti-mTOR, -AMPK, and -STAT3 antibodies of colon sections from the DSS vehicle and metformin groups. The score and positive area were determined. Data are means ± SEM of three replicates. *P < 0.05, **P < 0.01, ***P < 0.001.

Metformin induces SMILE in prostate cancer cells, thus suppressing the function of the androgen receptor (22). To investigate its effect on the SMILE level, intestinal HT-29 cells were treated with metformin and the level of phosphorylated STAT3 (Y705) was evaluated. Consistent with a prior report (22), metformin suppressed the phosphorylation of STAT3 and increased the protein level of SMILE in HT-29 cells (**Figure 1A**). To examine the effect of metformin on induction of SMILE *in vivo*, it was administered to DSS-induced colitis mice. Consistent with our previous work (23), metformin improved the loss of body weight and shortening of colon length in DSS-induced colitis mice compared to vehicle-treated DSS-induced colitis mice (**Figures 1B, C**). Metformin also increased the level of SMILE and suppressed that of mTOR and STAT3 in colon tissue of DSS-induced compared to vehicle-treated DSS-induced colitis mice (**Figure 1D**).

### Overexpression of SMILE Ameliorates DSS-Induced Colitis

**Figure 2:**
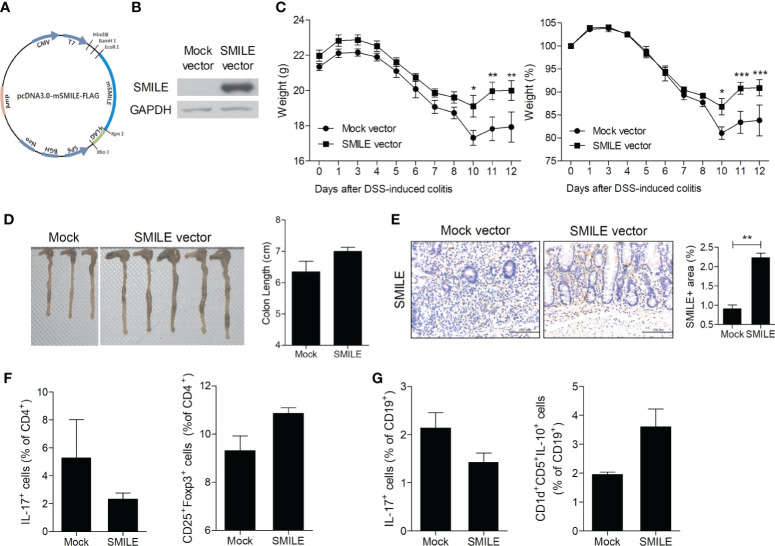
SMILE overexpression vector reduced the susceptibility of mice to DSS-induced colitis. **(A)** Vector map showing restriction sites and the SMILE site. **(B)** Lysates of HT-29 cells transfected with MOCK and SMILE overexpression vectors were analyzed for SMILE protein by western blotting with an anti-SMILE antibody. **(C)** Changes in body weight (n = 5 per group). **(D)** Macroscopic images of the colon and colon lengths. **(E)** Colon tissue stained with an anti-SMILE antibody. Representative images are shown. Bars are percentage positive areas per field. **(F)** Th17 and Treg cells and **(G)** IL-17- and IL-10-producing B cells in MLNs by flow cytometry. Data are means ± SEM of two replicates. *P < 0.05, **P < 0.01, ***P < 0.001.

**Figure 3:**
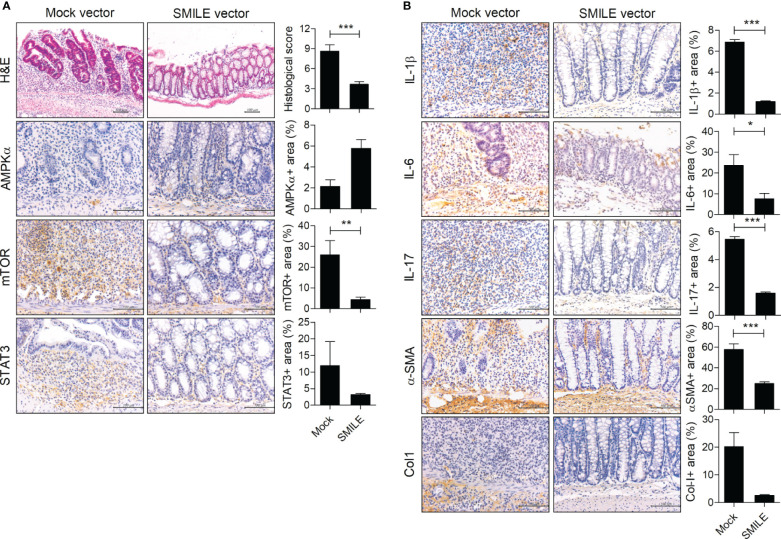
SMILE decreases inflammation and fibrosis in DSS-induced colitis. H&E and IHC staining of the colon of C57BL/6 mice with DSS-induced colitis and administered SMILE vector (100 μg/mL) (n = 5 per group). Representative images are shown. **(A)** H&E staining and IHC with anti-mTOR, -AMPK, and -STAT3 antibodies. **(B)** IHC with anti-IL-1β, -IL-6, -IL-17, -α-SMA, and -collagen-1 (Col1) antibodies. Bars are percentages of positive areas per field. Data are means ± SEM of two replicates. *P < 0.05, **P < 0.01, ***P < 0.001.

To determine whether overexpression of SMILE ameliorates IBD, we constructed a SMILE vector and evaluated its overexpression in HT-29 cells (**Figures 2A, B**). Injection of the SMILE vector prevented the loss of body weight and the decrease in colon length in DSS-induced colitis mice compared to mock-treated DSS-induced colitis mice (**Figures 2C, D**). SMILE enhanced SMILE vector treated colon tissue compared to mock-treated (**Figure 2E**). To determine whether overexpression of SMILE affects effector T-cell populations, we examined the number of Th17 and Treg cells as well as IL-17-producing and IL-10-producing B cells in the MLNs of DSS-induced colitis mice treated with SMILE or mock vector by flow cytometry. The number of Th17 cells and IL-17-producing B cells in the MLNs was reduced but the number of Treg and B10 cells was increased in mice with SMILE overexpression compared to mock vector-treated DSS-induced colitis mice, although there was no significance (**Figures 2F, G**). Next, the colon sections were subjected to IHC staining. The colon tissue from SMILE overexpressing DSS-induced colitis mice exhibited higher levels of SMILE and AMPK and lower levels of mTOR and STAT3 than that of mock vector-treated mice (**Figure 3A**). Administration of SMILE vector suppressed the levels of the proinflammatory cytokines IL-1β, IL-6, and IL-17 compared to mock vector-treated mice (**Figure 3B**). Furthermore, expression of α-SMA and collagen-1, markers of fibroblast differentiation, was decreased in colon tissues from SMILE overexpressing DSS-induced colitis mice compared to mock vector-treated mice (**Figure 3B**).

### DSS-Induced Colitis Is Ameliorated in SMILE Transgenic Mice

**Figure 4:**
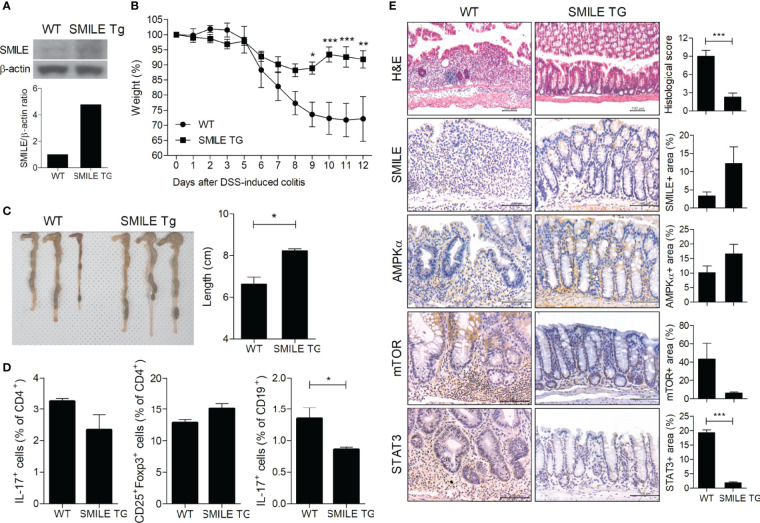
Therapeutic effect of SMILE in transgenic mice. **(A)** Transgenic mice overexpressing SMILE were generated on a C57BL/6 background. **(B)** WT and SMILE TG mice were treated with DSS and body weight was measured as a percentage of that at baseline (n = 5 per group). **(C)** Representative macroscopic images and colon length of WT and SMILE TG mice. **(D)** MLN cells analyzed by flow cytometry. **(E)** Colon tissue of WT and SMILE TG mice was subjected to H&E and IHC with anti-SMILE, -mTOR, -AMPK, and -STAT3 antibodies. Data are means ± SEM of two replicates. *P < 0.05, **P < 0.01, ***P < 0.001.

To confirm the therapeutic effect of SMILE, we induced DSS-induced colitis in WT and SMILE transgenic mice (**Figure 4A**). DSS-induced SMILE transgenic colitis mice showed recovery of body weight loss and colon length shortening compared to wild-type DSS-induced colitis mice (**Figures 4B, C**). The numbers of Th17 cells and IL-17-producing B cells from MLNs were lower and that of Tregs was higher in DSS-induced SMILE transgenic colitis mice compared to wild-type DSS-induced colitis mice (**Figure 4D**). The colon tissue from DSS-induced SMILE transgenic colitis mice showed increased levels of SMILE and AMPK and decreased levels of mTOR and STAT3 compared to the colon tissue of wild-type DSS-induced colitis mice (**Figure 4E**).

### Decreased SMILE Expression Was Restored by Metformin in PBMCs From Patients With UC

**Figure 5:**
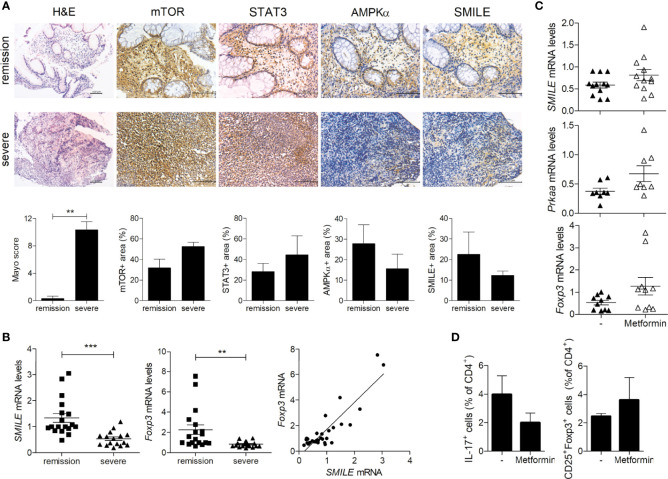
Regulation of SMILE expression in the colon of patients with UC. **(A)** Colon tissues were evaluated by H&E staining and Mayo scoring. Representative images of IHC with anti-mTOR, -STAT3, -AMPK, and -SMILE antibodies. Bars are percentages of positive area per field. **(B)** Fold changes in the SMILE and FOXP3 mRNA levels in UC PBMCs by real-time PCR (n = 9 per group). Correlation between SMILE and FOXP3 levels. **(C)** mRNA levels of SMILE, PRKAA, and FOXP3 in UC PBMCs treated with metformin (1 mM) for 48 h by real-time PCR. **(D)** Th17 and Treg cells in the PBMCs of metformin (1 mM)-treated patients with UC under Th17-polarizing condition for 72 h. Data are means ± SEM. **P < 0.01, ***P < 0.001.

We investigated SMILE expression in the colonic tissues of patients with UC. In tissue from patients with UC and severe inflammation, we observed decreased levels of SMILE and AMPK and an increased level of mTOR compared to patients with UC in remission (**Figure 5A**). The mRNA level of SMILE and Foxp3 was lower and that of SMILE was positively correlated with Foxp3 in PBMCs from patients with UC with severe inflammation compared to those in remission (**Figure 5B**). Next, PBMCs from patients with UC with severe inflammation or in remission were stimulated with metformin and SMILE, AMPK, and Foxp3 levels were assayed. Metformin increased the mRNA levels of SMILE, AMPK, and Foxp3 in PBMCs from patients with UC (**Figure 5C**). Furthermore, metformin reduced the number of Th17 cells among PBMCs from patients with UC, although there was no statistical significance (**Figure 5D**).

## Discussion

We investigated the mechanism by which SMILE suppressed the development of DSS-induced colitis. Metformin, an anti-diabetic drug and an inducer of AMPK, upregulated the level of SMILE in HT-29 human intestinal epithelial cells and the number of SMILE-expressing cells in colonic tissues from DSS-induced colitis mice compared with vehicle-treated DSS-induced colitis mice. Overexpression of SMILE reduced the severity of DSS-induced colitis and decreased colitis-associated intestinal fibrosis in the colon compared to mock vector. The expression of SMILE and Foxp3 was downregulated and that of SMILE was positively correlated with Foxp3 in PBMCs from patients with UC with an inflamed mucosa. Metformin increased the levels of SMILE, AMPK, and Foxp3 and decreased the number of Th17 cells among PBMCs from patients with UC.

AMPK, a serine/threonine kinase, triggers a metabolic switch from ATP consumption to ATP generation (24). AMPK inhibits inflammatory signaling pathways in various types of cells and tissues (25, 26). 5-Aminoimidazole-4-carboxamide ribonucleoside, an agonist of AMPK, ameliorates the severity of inflammatory diseases by reducing nuclear translocation of NF-κB and thus inhibiting the production of inflammatory cytokines in experimental autoimmune encephalomyelitis and IBD (25, 27). Furthermore, it suppresses lipopolysaccharide-induced TNFα production by blocking phosphatidylinositol 3-kinase (PI3K)/Akt activation in murine macrophages (28). We have shown that metformin, an AMPK activator, ameliorates the progression of IBD by suppressing STAT3 phosphorylation and modulating the balance between Th17 cells and Tregs (23). We confirmed the therapeutic effect of metformin on DSS-induced colitis in a murine model and found an increased level of SMILE in intestinal tissue. Moreover, metformin increased the SMILE protein level in human colon epithelial cells. This could explain the suppressive effect of metformin on the function of the androgen receptor in prostate cancer cells (22). In addition, epigallocatechin-3-gallate, a major component of green tea, and curcumin, a natural polyphenolic compound, trigger SMILE expression by inducing FoxO1 in hepatocytes (29) and *via* AMPK signaling (30), respectively.

SMILE is a multifunctional transcription factor that reduces hyperglycemia induced by CREB/CRTC2 signaling (31) and represses adipogenesis by regulating peroxisome proliferator-activated receptor γ (PPARγ) (32). CREBZF, the long form of SMILE, inhibits the ERK1/2 and mTOR signaling pathways by phosphorylating ERK1/2 and p70 S6 kinase (S6K1) and activates autophagy by increasing the LC3-II level in ovarian granulosa cells (33). Moreover, CREBZF stabilizes and activates the tumor suppressor p53 and protects against ultraviolet-induced cell death (34). However, few studies have focused on the role of SMILE in inflammatory diseases, including IBD. Here, we found that overexpression of SMILE improves weight reduction and intestinal shortening as well as fibrosis in DSS-induced colitis mice compared to control mice. SMILE downregulated mTOR-STAT3 signaling and upregulated the level of AMPK in colon tissue and reduced IL-17 production by splenic lymphocytes from DSS-induced colitis mice compared to control mice. Notably, PBMCs showed suppressed SMILE and Foxp3 expression, and SMILE expression was positively correlated with that of Foxp3 in patients with UC with severe inflammation compared to those in remission. mTOR and PI3K/Akt are key factors in the differentiation of Th17 cells (35). p53 exerts an anti-inflammatory effect in autoimmune diseases such as rheumatoid arthritis and modulates the balance between Th17 and Treg cells by directly binding STAT3 (36). Our results suggest that SMILE can ameliorate inflammatory diseases by regulating pathogenic T cells *via* mTOR and p53. Metformin increased the levels of SMILE, AMPK, and Foxp3 but decreased the number of Th17 cells in PBMCs from patients with UC with severe inflammation. Further studies are needed to identify the molecular mechanism underlying the role of SMILE in IBD. Overall, our data suggest that SMILE has therapeutic potential for IBD.

## Data Availability Statement

The datasets presented in this study can be found in online repositories. The names of the repository/repositories and accession number(s) can be found in the article/supplementary material.

## Ethics Statement

The studies involving human participants were reviewed and approved by Institutional Review Board of Seoul St. Mary’s Hospital. The patients/participants provided their written informed consent to participate in this study. The animal study was reviewed and approved by Department of Laboratory Animals, Institutional Animal Care and Use Committee of the School of Medicine, the Catholic University of Korea.

## Author Contributions

Conception and design of study: SY, J-SP, S-JK, B-IL, S-HP, M-LC. Acquisition of data: SY, S-HH, K-HC, JC, S-JK. Analysis and interpretation of data: SY, J-SP, HN, K-HC, JC, JJ. Drafting the article: SY, J-SP, M-LC. Revising the article critically: J-SP, S-JK, B-IL, M-LC. All authors contributed to the article and approved the submitted version.

## Funding

This work was supported by the National Research Foundation of Korea(NRF) grant funded by the Korea government(MSIT) (No. NRF-2020R1F1A1074916) and by a grant of the Korea Health Technology R&D Project through the Korea Health Industry Development Institute (KHIDI), funded by the Ministry of Health & Welfare, Republic of Korea (grant number HI20C1496).

## Conflict of Interest

The authors declare that the research was conducted in the absence of any commercial or financial relationships that could be construed as a potential conflict of interest.
